# Challenges in developing new tuberculosis vaccines

**DOI:** 10.1590/0074-02760240236

**Published:** 2025-06-09

**Authors:** Gabriela Sadigurschi, Maria Cristina Caetano Kuschnir, Ewerton Alves Portela dos Santos, Bruno Rangel Antunes da Silva, Celia Menezes Cruz Marques, Raissa Coelho de Andrade, Clarice Monteiro Vianna, Danillo Gonçalves de Barros, Mariana Torres Mazzi, Elvira Alonso Lago, Eliane Matos dos Santos, Maria de Lourdes de Sousa Maia

**Affiliations:** 1 Fundação Oswaldo Cruz-Fiocruz, Instituto de Tecnologia em Imunobiológicos/Bio-Manguinhos, Departamento de Assuntos Médicos, Estudos Clínicos e Vigilância Pós-Registro, Rio de Janeiro, RJ, Brasil; 2 Universidade do Estado do Rio de Janeiro, Hospital Universitário Pedro Ernesto, Rio de Janeiro, RJ, Brasil

**Keywords:** tuberculosis, vaccines, clinical trials, vaccine development, challenges, immunity

## Abstract

Tuberculosis (TB) is a preventable and curable disease caused by the bacillus *Mycobacterium tuberculosis*. In 2022, according to the World Health Organisation (WHO), TB was the second leading cause of death worldwide caused by a single infectious agent, after coronavirus disease (COVID-19). Brazil is ranked among the 30 countries with the highest TB burden. Currently, the neonatal Bacillus Calmette-Guérin (BCG) is the only vaccine against TB and offers significant efficacy against disseminated and meningeal disease in children. However, BCG has a limited efficacy in preventing adult-type cavitary TB, reinforcing the need for a new effective vaccine against pulmonary TB. There are currently over 22 TB vaccines under evaluation in clinical trials worldwide. Despite significant advancements, several challenges persist in developing and producing an effective TB vaccine. These include understanding the immune mechanisms that confer protection against *M. tuberculosis*, identifying immune correlates of protection, defining immune responses in BCG-vaccinated individuals, establishing efficacy endpoints for TB vaccine trials, and ensuring vaccine safety and effectiveness in individuals with human immunodeficiency virus (HIV), among other obstacles. Therefore, this study aims to explore the key obstacles in developing new TB vaccines and potential strategies to overcome them.

Tuberculosis (TB) is a preventable and curable disease caused by the bacillus *Mycobacterium tuberculosis*. Transmission occurs through prolonged contact with aerosols generated by infected individuals, usually through coughing and sneezing, especially in poorly ventilated and crowed places. In 2022, according to the World Health Organisation (WHO), TB was the second leading cause of death worldwide due to a single infectious agent, after coronavirus disease (COVID-19). Of the total number of people who develop TB each year, around 90% are adults. The disease primarily affects the lungs causing pulmonary TB, but it can also manifest as extrapulmonary TB, involving lymph node, pleura and other organs.^1^

Currently, the standard TB treatment regimen has a course of six months and includes the drugs isoniazid, rifampin, pyrazinamide, and ethambutol. Around 85% of patients can be cured; however, without treatment, the mortality rate is high, reaching up to 50%. Another concerning issue is the increasing number of drug-resistant TB cases. In 2022, it was estimated that more than 410,000 people worldwide developed drug-resistant TB.^1^

According to the WHO report published in 2023, Brazil is ranked among the 30 countries with the highest TB burden. The BRICS group (Brazil, Russia, India, China and South Africa) account for 38% of TB cases worldwide. In 2023, Brazil recorded 84,000 new TB cases and more than 5,900 deaths. People in vulnerable situations are at greater risk of illness, including indigenous people, people living with the human immunodeficiency virus (PWHIV), people deprived of liberty, people living on the streets and immigrants.^1^ ^,^ ^2^

Regarding immunisation, the neonatal Bacillus Calmette-Guérin (BCG) vaccine is a live attenuated vaccine form of *M. bovis*, first administered to humans in 1921. Currently, BCG is the only approved vaccine against TB and is highly effective in preventing disseminated and meningeal disease in children. Reports have also shown that BCG provides significant protection against leprosy and Buruli ulcer.^3^ ^,^ ^4^ A meta-analysis by Martinez et al. highlighted the importance of infant BCG vaccination, particularly for young children at high risk of TB. However, BCG has limited efficacy in preventing adult-type cavitary TB, reinforcing the need for a new, effective vaccine against pulmonary TB.^5^

Given the impact of this disease, global targets for TB have been set in the United Nations Sustainable Development Goals (SDG), the WHO End TB Strategy and the political declaration of the United Nations High-Level meeting on TB, reviewed in 2023. One of the SDGs includes the goal of ending TB globally by 2030. Additionally, the WHO End TB strategy aims to eliminate the global TB epidemic by 2035, with a target of reducing global TB incidence by 90% and mortality rates by 95% compared to 2015.^6^ ^,^ ^7^ In Brazil, the goals include reducing TB incidence to fewer than 10 cases per 100,000 inhabitants and reducing TB deaths to fewer than 230 deaths. In 2023, the WHO Director-General launched the TB Vaccine Accelerator Council to facilitate the development of new TB vaccines.^8^

The WHO has developed a document outlining the preferred characteristics for TB vaccines targeting adolescents and adults. Some of these specifications include an efficacy of 50% or greater in preventing bacteriologically confirmed pulmonary TB and protection for individuals with and without evidence of latent infection across different geographic regions. For infants and young children, the vaccine should prevent TB disease, including severe and disseminated TB, tuberculous meningitis and pulmonary TB. Efficacy should be at least 80% compared to baseline incidence or superior to BCG.^9^

The ideal TB vaccine should offer protection for ten years or more after primary immunisation. Safety must be ensured, particularly for high-risk groups such as individuals living with HIV/AIDS or other immunodeficiencies, the elderly, pregnant and breastfeeding women. Furthermore, vaccines should provide protection against drug-sensitive TB and drug-resistant TB.^9^ Cost-effectiveness must be favourable, and pricing should not be a barrier to access, particularly in low- and middle-income countries.^10^

A study by Knight et al. using mathematical modelling found that between 2024 and 2050, a vaccine targeting adolescents and adults could have a greater impact than one aimed at infants. In low-income countries, a vaccine with a 10-year duration and 60% efficacy targeted at adolescents and adults could prevent 17 million TB cases by 2050. In contrast, if targeted at infants, the vaccine could prevent 0.89 million TB cases. Consequently, while an improved childhood vaccine may be beneficial in the long term, in the short term, a vaccine for adolescents and adults is expected to have a more immediate and significant impact.^11^

According to Junqueira et al., over 22 TB vaccines are currently being evaluated in clinical trials worldwide.^12^ Despite the progress in vaccine development, several challenges remain, including understanding the immune mechanisms of TB protection, defining immune correlates of protection*,* determining the immune profiles of BCG-vaccinated individuals, establishing efficacy endpoints for TB vaccine trials and ensuring the development of a safe and effective vaccine for people with HIV. This study aims to elucidate challenges involved in the development of new TB vaccines.

Understanding the immune process of TB protection remains a significant challenge. The immune response to TB is not fully understood, given the complexity of TB-host interactions. An optimised vaccine should prevent *M. tuberculosis* from entering host cells and enhance both innate and adaptive immune responses. The primary goal of TB vaccination is to create a robust and lasting immune memory to protect against *M. tuberculosis*.^13^ It is widely accepted that TB immunity relies primarily on granuloma formation, which is driven by cell-mediated immunity. In contrast, humoral immunity is generally thought to play a minimal role in providing protection.^14^ However, recent studies suggest that humoral immunity may also contribute significantly to defence against *M. tuberculosis* infection.^15^

Some of the immune mechanisms in TB vaccines involves CD4 + T cells, CD8 + T cells, B cells and other immune cells such as NK cells and unconventional T cells. The CD4+ T cells represent the main mechanism to control TB.^16^ Moreover, the antigen-specific natural T cell response to *M. tuberculosis* is highly diverse, as CD4+ T cells recognise numerous antigens, each with distinct expression patterns at different stages of infection. Therefore, a great number of antigens are necessary to cover 80% of CD4 T cell responses. The mechanisms by which *M. tuberculosis* evades T cell-mediated immunity are not yet fully understood.^17^ This factor is relevant considering that vaccines platforms based on few antigens may not induce a sufficient immune response, turning necessary to choose multiple antigens with diverse components to develop effective vaccines.^16^

Currently, the human immune correlates of protection against *M. tuberculosis* infection and TB disease are not completely comprehended. Studies have shown an association between IFNγ-producing BCG-specific T cells and a reduced risk of TB disease in vaccinated infants.^18^ ^,^ ^19^ In recent studies, *M. tuberculosis*-specific immunoglobulin M (IgM) has been identified as a potential immune marker for protection in rhesus macaque vaccinated intravenously with BCG.^20^ However, relying on a single immune marker is insufficient to accurately predict the protection conferred by BCG vaccination. Therefore, it is essential to assess multiple host factors to identify more reliable correlates of protection.

Another significant challenge is the varied immunisation status stemming from previous BCG vaccinations, as the immune profiles of vaccinated individuals and their infection backgrounds are diverse. The widespread use of BCG, the non-tuberculous mycobacteria (NTM) infections in countries with high burden of TB and the increasing number of individuals with latent TB (LTBI) turns this landscape more complex.^21^ It was shown that LTBI population exhibit distinct antibody profiles compared to those with active TB, which could help differentiate between these two disease states. These differences are also linked to Fc receptor functions, and the antibodies predominant during latent infection are associated with better control of replicating intracellular bacteria. This data reinforces the diversity of the population’s immune profile associated with previous exposure to *M. tuberculosis*, which becomes relevant data in the development of TB vaccines.^15^ Also, vaccine TB candidates with platforms such as protein/adjuvant subunit vaccines and viral vector vaccines are designed to be given as a booster vaccine following BCG vaccination. Thereby, the efficacy of these vaccines depends on whether BCG vaccination induced an effective primary immune response. Therefore, it’s essential to accurately differentiate the diverse immunisation status to optimise vaccination effectiveness.^22^

Another relevant aspect that a new TB vaccine should incorporate is the ability to induce a mechanism known as “trained immunity”, similar to BCG. This process involves epigenetic and metabolic reprogramming of innate immune cells, leading to enhanced surface marker expression and cytokine responses upon secondary stimulation.^23^ A study conducted by Tarancón et al. demonstrated that MTBVAC, a live attenuated vaccine derived from the human pathogen *M. tuberculosis* through genetic modification, is also capable of inducing trained immunity *in vitro*.^24^

Determining the efficacy endpoint for TB vaccine trials is a crucial aspect of new TB vaccine development. In preclinical animal models, vaccine effectiveness is typically assessed through improvements in histopathological damage, reductions in bacterial counts in organs, and survival time. However, in human clinical trials, TB incidence is used as the primary endpoint, highlighting a key difference between preclinical and clinical trials in evaluating vaccine effectiveness.^21^

The gold standard to measure TB vaccine efficacy is the prevention of microbiologically confirmed TB disease, which is usually measured among adolescents and adults using mycobacterial growth indicator tube (MGIT) liquid culture from respiratory specimens. However, limitations of this method are the need for a central laboratory, variable contamination rate as the analysis is largely laboratory-dependent and lower performance in TB paucibacillary population such as children and PWHIV. Another faster and more cost-effective alternative used in TB vaccine trials is evaluating the prevention of clinically asymptomatic *M. tuberculosis* infection, using gamma release assays (IGRA) from the different QuantiFERON (QTF) generations (QTF-Gold, QTF-TB Gold in Tube or QTF-TB Gold Plus test, by Qiagen).^25^ Measuring incident *M. tuberculosis* infection through seroconversion of IGRA is particularly relevant, as individuals who have recently acquired *M. tuberculosis* are at a higher risk of developing TB disease. However, this approach has some limitations, including the unclear significance of IGRA in TB disease protection and the fact that some individuals with culture-confirmed TB disease remain IGRA negative, suggesting that not all individuals seroconvert at the time of infection. This highlights the need for new, more effective tests to detect established *M. tuberculosis* infection and further research to clarify the clinical significance of IGRA seroconversion.^25^ ^,^ ^26^

Another important consideration in TB vaccine development is the use of controlled human infection models (CHIMs), as *M. tuberculosis* cannot be used to challenge humans. A study by Harris et al. investigated intradermal BCG challenge as a substitute for *M. tuberculosis* challenge in healthy individuals and found that those previously vaccinated with BCG exhibited some degree of protective immunity.^27^ Additionally, Davids et al. explored a lung-oriented CHIM using live BCG, demonstrating that bronchoscopic instillation of live BCG into the lungs of healthy participants is both safe and feasible in a TB-endemic setting.^28^ Regarding animal models, there is a lack of suitable models for TB vaccine research. Studies have shown that *M. tuberculosis* infection and disease manifestations vary across species, suggesting that differences between humans and animals may significantly impact the predictive accuracy of these models. The most used animal model for evaluating TB vaccine candidates is the mouse model, followed by guinea pigs, rabbits, cattle, and non-human primates (NHPs).^29^ Mouse models offer advantages such as affordability, high viability, and the widespread availability of detection reagents. However, they are limited by their lack of natural susceptibility to mycobacterial infection.^30^ Additionally, a significant difference exists between the controlled exposure in animal models and natural human infection. NHPs are the most effective models for studying human TB due to their genetic similarities to humans. However, their use is restricted by substantial economic and ethical considerations, particularly given the large number of TB vaccine trials currently underway.^31^

Genetic variability between individuals also represents a challenge in developing a preventive TB vaccine. Substantial variability in response to the current BCG vaccine is attributed to individual differences such as age, sex, method of vaccination, nutritional status, among others. A study conducted by Finan et al. found that cytokine responses to mycobacterial antigens in BCG-vaccinated infants are heterogeneous and there is significant inter-individual variation.^32^ Future TB vaccine candidates must consider the various immune mechanisms and develop strategies to identify different subsets of individuals, which will help determine the most effective vaccine type or adjuvant combination to use.^33^

Evaluating the safety and efficacy of TB vaccines in PWHIV is another crucial factor, as this population is at a higher risk of developing TB disease and experiencing more severe outcomes. The current recommendation from the Brazilian Ministry of Health for newborns exposed to HIV is to administer BCG at birth or as early as possible, as well as to administer it to children who arrive at the health service weighing more than 2 kg, asymptomatic and without signs of immunodeficiency. However, it is not recommended to administer BCG to children infected with HIV from the age of five onwards, even if they are asymptomatic and have no signs of immunodeficiency.^34^ According to a study published by Miner et al., nine completed studies involving six TB vaccine candidates have included PWHIV. These candidates include two viral vectored vaccines (MVA85A, Aeras-402), two subunit vaccines (H1:IC31, M72/AS01E), and two whole-cell inactivated bacterial vaccines (RUTI, *M. obuense*). Overall, the study found that TB vaccines in PWHIV are safe, induce cellular immunity, and exhibit variable durability. Additionally, several trials are underway to evaluate the following vaccines in PWHIV: VPM1002, ID93/GLA-SE, DAR-901, and MTBVAC.^35^

Other lines of research to improve TB vaccination are directed towards addressing mucosal immunisation and alternative routes of TB vaccination. In this context, inhalable TB vaccines offer the potential to mimic the natural route of *M. tuberculosis* infection and provide durable mucosal immune responses.^36^ It was found that the intranasal administration of whole cell inactivated MTBVAC HK vaccine to previously BCG vaccinated mice substantially improved the protective efficacy conferred by subcutaneous BCG only.^37^ A phase 1 clinical trial compared the safety and immunogenicity of aerosolised and intradermally administered MVA85A in healthy BCG-vaccinated adults in the UK and found that both administration routes were well tolerated, and immunogenic.^38^ Another study found that inhaled ChAdOx1-85A vaccine was well-tolerated, effectively inducing lung mucosal and systemic Ag85A-specific T-cell responses.^39^

The lack of investment in TB development remains a significant barrier. According to the report from the 2022 Global Forum on Tuberculosis Vaccines held in Toulouse, it is necessary to address the chronic underfunding for TB vaccine research and development with innovative funding mechanisms, stronger political leadership, and support from all sectors, including public, private, and philanthropic organisations.^40^ In 2019, only $116 million was invested in TB vaccines, compared to $28 billion allocated for COVID-19 diagnostics, therapeutics, and vaccines. This persistent underfunding underscores the inequalities surrounding TB, a disease that primarily affects low- and middle-income countries rather than high-income nations.^41^

In conclusion, the development of a new TB vaccine has proven to be extremely challenging due to several factors such as the incomplete understanding of *M. tuberculosis* protective immunity, the absence of well-defined correlates of immune protection, the lack of suitable animal models and the diverse and specific population groups exposed to *M. tuberculosis* such as PWHIV, children and LTBI population. Although TB vaccine development is already underway, it is essential to increase investments from all sectors such as public, private, and philanthropic organisations.
